# Quantitative data management in quality improvement collaboratives

**DOI:** 10.1186/1472-6963-9-175

**Published:** 2009-09-26

**Authors:** Mireille van den Berg, Rianne Frenken, Roland Bal

**Affiliations:** 1 XXscience, Koningsdam 1, Rotterdam, The Netherlands; 2 Healthcare Inspectorate of the Netherlands, Parnassusplein 5, The Hague, The Netherlands; 3 Dept. of Health Policy and Management, Erasmus University Medical Centre, P.O. Box 1738, 3000 DR Rotterdam

## Abstract

**Background:**

Collaborative approaches in quality improvement have been promoted since the introduction of the Breakthrough method. The effectiveness of this method is inconclusive and further independent evaluation of the method has been called for. For any evaluation to succeed, data collection on interventions performed within the collaborative and outcomes of those interventions is crucial. Getting enough data from Quality Improvement Collaboratives (QICs) for evaluation purposes, however, has proved to be difficult. This paper provides a retrospective analysis on the process of data management in a Dutch Quality Improvement Collaborative. From this analysis general failure and success factors are identified.

**Discussion:**

This paper discusses complications and dilemma's observed in the set-up of data management for QICs. An overview is presented of signals that were picked up by the data management team. These signals were used to improve the strategies for data management during the program and have, as far as possible, been translated into practical solutions that have been successfully implemented.

The recommendations coming from this study are:

From our experience it is clear that quality improvement programs deviate from experimental research in many ways. It is not only impossible, but also undesirable to control processes and standardize data streams. QIC's need to be clear of data protocols that do not allow for change. It is therefore minimally important that when quantitative results are gathered, these results are accompanied by qualitative results that can be used to correctly interpret them.

Monitoring and data acquisition interfere with routine. This makes a database collecting data in a QIC an intervention in itself. It is very important to be aware of this in reporting the results. Using existing databases when possible can overcome some of these problems but is often not possible given the change objective of QICs.

Introducing a standardized spreadsheet to the teams is a very practical and helpful tool in collecting standardized data within a QIC. It is vital that the spreadsheets are handed out before baseline measurements start.

## Background

Quality collaboratives have gained in attention since the formulation of the "quality chasm" by the US Institute of Medicine [1] and its spread across the Western world. The Breakthrough method developed by the Institute of Health Improvement has been one of the major instruments put to use in such collaboratives. Quality improvement collaboratives (QICs) are seen as a means to spread evidence-based practices quickly across care organizations, as there is some evidence that the integration of quality instruments leads to synergistic effects [2]. As noted in the literature, however, hardly any evidence exists as of yet to the effectiveness of quality collaboratives in bridging the quality chasm: do collaboratives indeed lead to better care? Are they doing this in an efficient manner? Questions like these have hardly been systematically addressed [3,4]. There are some indications that a significant publication bias exist, and most studies are methodologically weak, e.g. relying on self-reporting [5]. The few systematic studies that have been done are inconclusive as some show no improvement [6,7] whereas others show significant improvements [8]. A recent review showed some positive effects, but the study base for this review was very limited [9]. These mixed effects have been attributed to several factors, i.e. differences in external context of care providers, cultural aspects, team functioning, availability of resources [10]. Further evaluations of quality collaboratives are in dire need, if only to bridge the "apparent inconsistency between widespread belief in and use of the QIC method and the available supporting evidence" [3].

In large and complex organizations such as most quality collaboratives, data management can be a critical factor in communicating results, both within the collaborative as outside. However, data collection and management within a QIC poses significant challenges and dilemmas, mainly arising from the possible contrast between the objective of the collaborative to improve care and the need to gather reliable data from the evaluator's perspective [11,12]. For any evaluation to succeed, data collection on interventions performed within the collaborative and outcomes of those interventions is crucial. Getting enough data from QICs for evaluation purposes, however, has proved to be difficult (e.g. [10] report to have a response rate of some 50%). This paper provides a retrospective analysis on the process of quantitative data management in a Dutch QIC, the so-called Faster Better pillar three program (FB p3) in acute care hospitals. From this analysis general failure and success factors will be identified. Below, these dilemmas as met in the FB p3 program are analyzed and interventions to solve them within this program described.

### Faster Better

**Figure 1:**
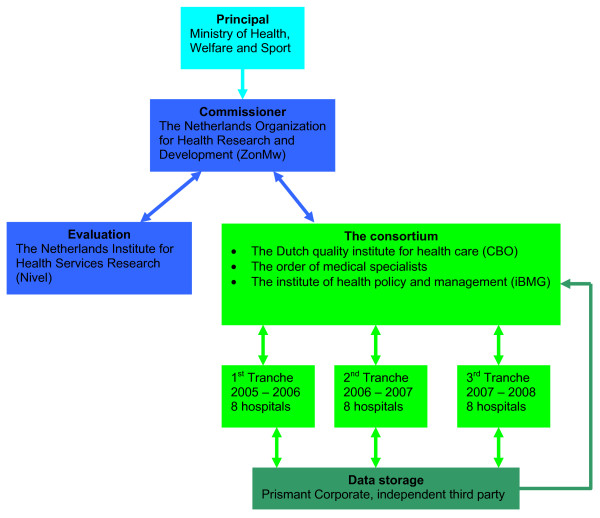
**Parties involved in the FBp3 program**.

The Dutch Faster Better program is used as a case for this article. In October 2004 the Faster Better Program pillar 3 (FB p3) was launched in eight Dutch hospitals. The 'pillar 3', distinguishes the program from the 1^st ^pillar in which CEO's from multinational businesses were asked to reflect on developments in health care, and the 2^nd ^pillar in which the Healthcare Inspectorate developed and implemented performance indicators for hospitals. The Ministry of Health, together with the main associations of hospitals, medical professionals and patients, overlooked the whole program. The pillar 3 program on which is reported here was performed by a consortium of the Dutch quality institute for health care CBO, the Order of Medical Specialists and the institute of Health Policy and Management. It had to inform the commissioner and was therefore responsible for data management. The program was independently evaluated by the Netherlands Institute for Health Services Research (Nivel). Figure 1 provides insight in the position of the involved parties.

**Figure 2:**
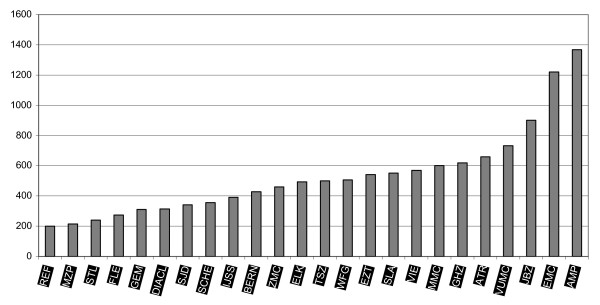
**Number of beds in the FBp3 hospitals**.

The aim of this quality improvement program was to help health care providers in the field improve their performance, starting with hospitals and primary health care. In three cohorts of 8 hospitals each lasting two years a total of 24 hospitals (20% of the total amount of Dutch hospitals in the public sector) were enrolled in a quality improvement program aiming at patient safety and patient logistics (see appendix I). Participating hospitals were a mix of academic and general hospitals. Both relatively small (200 beds) and large (1368 beds) hospitals were involved in the FB p3 program (see Figure 2 for the number of beds in each of the participating hospitals).

The general goal of the FB p3 program was inducing quality improvement in the participating hospitals by implementing best practices, sharing knowledge and securing and documenting successes. The FB p3 program aims at improving on safety, logistics, leadership and patient centered working, increasing transparency of health care in The Netherlands. Sharing successes and best practices with other hospitals, not participating in the FB p3 program, should eventually lead to widespread implementation of quality improvement interventions [13,14].

In order to secure these goals breakthrough projects on selected themes were organized and supported both by nationally operating project leaders and, in the hospitals, by hospital advisers. Hospitals, though left free to some extent on the internal organization of the program, were expected to appoint a 'project coordinator' as well as project teams participating in the Breakthrough collaboratives. The participating teams were brought together for at least three learning sessions, at the start of the project, half way the project and at the finish of the project. Furthermore, the project leader delivered consultation on a project level to the teams in-person. Hospitals could rely on the hospital adviser for more practical issues. For hospital CEOs and clinical leaders, a 'leadership' program was developed.

### Data management

Within the FBp3 program data management served multiple purposes. On team level the data was used in order to provide feedback to motivate and inform the teams as part of the Breakthrough method. On program level data was used to monitor the larger program. External evaluators used the database for evaluation of program outcomes at the patient level, but collected their own (survey and interview) data for other parts of the evaluation [15]. Because of the introduction of both the performance indicators to control and improve quality of health care in the Netherlands (the Faster Better pillar 2 program) by the Dutch Health Care Inspectorate in 2003 and the introduction of a new health care financing system in 2006, it was virtually impossible to rely for evaluation on existing medical databases.

**Figure 3:**
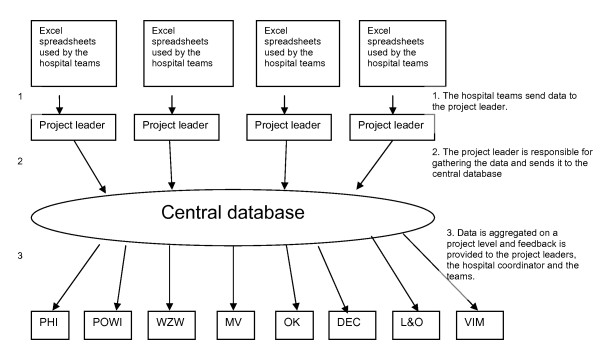
**Organization of data management within the FBp3 program**.

The consortium was responsible for data management. Data was gathered from all the participating teams. In order to define quantitative goals at least one key-indicator was defined for each QI project. This occurred in close consultation with the project leaders, which delivered the indicator set to the hospital teams and provided them with training required for data gathering. These key-indicators provided standardized data that was essential to reduce measurement variability (see Appendix I for an overview of quantitative program goals). In order to minimize the administrative burden, it was essential to use existing databases as a source for these indicators as much as possible. Using existing databases can also help to avoid outcome attribution failure because data of non-participating hospitals can be used as control data (e.g. for cohort effects). Data was gathered by means of excel spreadsheets that were developed specifically for the teams. For each type of improvement target a spreadsheet was developed. By filling out the spreadsheets the teams received instant feedback on the indicators of their projects. On top of the feedback the teams received progress reports on project level and on the complete program every three months. The project leaders were responsible for gathering data from all hospital teams on their projects and reported this to the data management team. The data management team dealt mainly with the project leaders and never directly with the teams or the coordinators within the hospitals. Figure 3 provides insight in the organization of data management within the FB p3 program.

For monitoring progress a data warehouse was designed. The data warehouse construction for data management was chosen because of the complexity of the program. Within the FB p3 program 24 hospitals participated in three separate cohorts. These cohorts started in October 2004, October 2005 and the last one in October 2006. Each cohort lasted for two years. Each year newly formed multidisciplinary teams from different departments participated in one of the projects on patient safety and patient logistics. An overview of all projects is given in Table 1. Within each cohort of eight hospitals a minimum of 1 and a maximum of 31 teams participated in 7 different projects. In each project a number of indicators was measured that gave information on aspects of the project representative for the progress of the whole project. In the fourth year there were up to 183 different teams participating in de FB p3 program. In total, 515 teams participated in the program.

**Table 1:** Teams participating in the QI projects at any time in the first four years of the FB p3 program

	**1^st ^year**		**2^nd ^year**		**3^rd ^year**		**4^th ^year**	
**Project**	**Y1C1**		**Y2C1 & YY1C2**		**Y2C2 & YY1C3**		**Y2C3**	
	**Hospitals (n)**	**Teams (n)**	**Hospitals (n)**	**Hospitals (n)**	**Hospitals (n)**	**Teams (n)**	**Hospitals (n)**	**Teams (n)**
Decubitus Ulcers	8	19	16	31	16	32	8	15
Medication	7		14		13		7	
safety		7		10		14		5
Postoperative		11		3		5		2
pain		1		4		4		3
Antibiotic Switch								
Blood								
transfusion								
Advanced Access	8	22	15	40	16	50	8	17
Process redesign	8	23	16	41	16	39	8	16
Postoperative wound infections	8	9	15	17	14	23	7	13
Operating Room OK	-	-	14	17	16	16	8	8
								
**Total**		92		163		183		77

### Data management protocol

The outline of the data warehouse used for data management was carefully described in a data management protocol with instructions on how the data should be handled. All parties were informed and asked to agree before data was gathered. The protocol contained information on the indicator-set, what they measured and the frequency with which data should be provided. Teams could add local indicators on the spreadsheets. However, these were not aggregated and analyzed by the data management team. Only the data manager could access the database and the involved parties could request for the aggregated data. The data was stored on a secure intranet network provided by Prismant Corporate, an independent third party known for storing medical data of all general and academic hospitals in The Netherlands. Prismant Corporate had no access to the data and was not involved in data management activities.

### Procedure

This paper is reporting on complications observed by the data management team in retrospective. It is based on progress reports of the data management project and notes of the monthly meetings in which data management was a returning subject. During the FB p3 program the data management team assessed together with the project leaders what factors caused the perceived problems in data management. Jot notes taken at these sessions have been analyzed for the purpose of this communication. In this communication an overview is presented of signals that were picked up by the data management team. These signals were used to improve upon the strategies for data management during the process of data gathering and have, as far as possible, been translated into practical solutions that have been successfully implemented. The main purpose of this was to adjust and improve the database in order to be able to report on the results of the FB p3 program.

### Data coverage

From Table 2 it is clear that in the 1^st ^year of the 1^st ^cohort (Y1C1) less than half of the expected data actually is present in the database. In December 2005 the first year was closed with an overview that was send not only to the participating teams, but also to the management of the participating hospitals. After this a secondary call for data went out after which the database contained nearly three quarters of the expected data. In May and June 2006 there was a call for data for the purpose of an interim score.

**Table 2:** Percentage data coverage (received files)

**Date**	**% Y1C1**	**% Y2C1**	**% Y1C2**	**%Y2C2**	**% Y1C3**
Start Y1C1					
November 2005	47				
Start Y2C1 & YY1C2					
December 2005	55				
January 2006	73.5				
18-05-06	85 baseline 82.5 follow-up	55 baseline	69.5 baseline		
06-06-06		59 baseline	73 baseline		
13-09-06		77.5 baseline	93 baseline		
22-09-06		85 baseline	93 baseline		
		36.5 follow-up	47 follow-up		
Start Y2C2 & YY1C3					
24-11-06		88 baseline	94 baseline		
		50 follow-up	63 follow-up		
08-12-06		87 baseline	97 baseline		
		53 follow-up	70 follow-up		
29-01-07		90 baseline	98 baseline		
		56 follow-up	81 follow-up		
15-04-07		90 baseline	98 baseline	71 baseline	51 baseline
		58 follow-up	82 follow-up		
25-05-07		90.7 baseline	99.1 baseline		
		57.1 follow-up	81.7 follow-up		
11-06-07				78 baseline	61.9 baseline
05-07-07				83.2 baseline 62.3 follow-up	85.6 baseline 37.7 follow-up
				83.2 baseline	87.5 baseline
31-08-07				72.9 follow-up	45.7 follow-up
03-09-07				83.2 baseline	88.4 baseline
18-09-07				76.8 follow-up	60.6 follow-up
04-10-07					
09-10-07				83.2 baseline 76.8 follow-up	91.2 baseline 68.3 follow-up
13-11-07				83.2 baseline 76.8 follow-up	91.2 baseline 69.2 follow-up
02-01-08				83.2 baseline 78.8 follow-up	91.2 baseline 76.0 follow-up
30-01-08				83.2 baseline 78.8 follow-up	91.2 baseline 76.0 follow-up
28-02-08*				83.2 baseline 80 follow-up	94.0 baseline 75.5 follow-up
20-03-08				83.2 baseline 80 follow-up	94.8 baseline 77.3 follow-up

*At the time of writing this paper, not all follow-up data was available.

It becomes clear from the data presented in Table 2 that problems with data management were manifest during Y1C1 and that measures taken thereafter solved the data management problem in large part. Most of the Y1C1 data was acquired in the years following the first year of the FB p3 program. Interpreting the Y1C1 data however remains problematic, especially with regard to follow-up measurements, because by the time this data was gathered most of the context information was lost.

A difficulty with the initially lacking data for Y1C1 was that all teams had been working with great enthusiasm for a whole year and there was no data to prove it by. These results lead to an introspective research in order to explain why participating teams appeared not to be able to provide the information they were requested. There were various explanations. These will be described and analyzed in this paper together with the measures to improve data management.

## Discussion

### Data sensitivity

The first factor that could explain reservations toward data management is that the involved parties were very cautious. The FB p3 program was launched in the context of a significant reform of the Dutch health care system [16,17], and was presented as part of that reform. At that time there was a great increase in attention for health care quality in The Netherlands. Most important developments in The Netherlands at the onset of the FB p3 program are the introduction of a new health care financing system, the diagnosis-related group as part of the upcoming introduction on regulated competition, which was introduced in 2006. Furthermore in 2003 the Dutch Health Care Inspectorate introduced a number of quality indicators to control and improve quality of health care in the Netherlands (the Faster Better pillar 2 program). At the start of the program, the first of the 'top 100' lists of hospital performance - based on healthcare inspectorate indicators [18] - were published which resulted in major public debates about both hospital performance and the legitimacy of such listings [19]. This created a climate in which central data management was considered a risk factor of being exposed.

Despite the data management protocol and even though the database was kept in a secure environment and all data traffic was password secured, this was not enough to prevent resistance. At different levels in the process there was great resistance to the principle of standardized data management. The suggestion that the gathered information would be used for benchmarking purposes raised a lot of critique from as well the participating hospitals as some of the partners of the consortium. The most fundamental worry was that, despite the data management protocol, the gathered data would not be secure and would be used for other purposes than described in the protocol. Some feared that they would not be able to publish their own data, whereas others were worried that there would be no restriction for third parties to publish all data. There was a great *lack of trust *among the involved parties with regard to data management.

Another problem was the suggestion that whenever a team would not be able to reach its goals, the results could be used against them, either by hospital management or by other stakeholders (e.g. insurance companies, politics, media). On the program level, this proved to be the case as well, when in 2006 the NIVEL, that was assigned to evaluate the program, published its report on the results of the first year of the program, accompanied by a press release that had as a title that "only 20% of Faster Better projects meet the target" [20]. Although in the body of the press release this heading was nuanced, it triggered significant discussion amongst the partners in the program.

### Action

The data management team invested a lot of time in communication. Meetings were scheduled at all levels in the FB p3 program. At the level of the consultants and the hospitals the procedures were explained and explicated. It was made clear how and why data was gathered and who had access to the database. This information was also accessible in the data management protocol. The experience was however that making an extra effort in explaining data management face to face created more trusts and willingness to cooperate. At the management level of the FB p3 program the data management team emphasized the importance of transparency and communication. It was necessary to create consciousness at all levels of the vital importance of a reliable database, both for learning within the collaborative as for program accountability. In the second and especially the third cohorts of hospitals entering the program, distrust towards data collection and management decreased. Apart from more intensive communication, also in the selection phase of the program, the longer experience with public disclosure of performance data might have attributed to this.

Furthermore, to insure absolute secure data handling the data was stored on a secure intranet network provided by an independent third party (Prismant Corporate). An important reason for storing the data with this third party organization was to gain trust within the hospitals. It was presumed that it would be easier for the hospitals to send their data to this third party organization then to a member of the Better Faster consortium, even if this was a university department. A member of the data management team held office at this organization twice a week. The password-secured spreadsheets were sent to an email address connected to the third party organization. The passwords were altered for each series. The flipside was that despite all these efforts there was a lot of unsecured data traffic. The main reason for this was that people thought the security measures were too extensive, they had problems remembering the password or they were not aware of the risks of unsecured data traffic by email. In 2007, Prismant stopped offering the service of storing data; at that point, trust in the data management project had increased and it was decided to build a secure server at the university department participating in the program.

### Political sensitivity

In line with the perceived sensitivity of the data it was felt that the comparability resulting from the central database would generate political sensitivities within the participating hospitals, especially with regard to some projects. For instance, earlier benchmarks (that were associated with the Faster Better program) around the performance of operating rooms (OR's) had generated a host of hospital-internal discussion. It appeared that some of the hospitals, also involved in the FB p3 program, were working with 50% overcapacity in the OR's: nearly half of the staff in these OR's where considered redundant by hospital management as a result of these benchmarks. Regarding the sensitivity of this outcome, working with centrally defined indicators in this sense was thought to jeopardize the ability of the program to get support from internal hospital teams.

### Action

As creating better efficiency in the hospitals was an explicit goal of the program, the anxiety at work floor levels of the discovery of overcapacity was understandable. Rather than focusing on discharging personnel however, the program was focusing on using efficiency gains by further investing in quality of care, raising hospital production and/or postponing capacity increases. This was made clear in an added project on creating business cases for the improvement projects and was discussed with hospital CEOs in the leadership program.

### Breakthrough method

Besides the sensitivity of using centrally registered results for data management purposes, there were two philosophies colliding. Whereas the data management within the FB p3 program aimed to gather as much as possible standardized data with regard to the main goals of the program, the methods used within the program were not fit for this strategy. The quality improvement interventions implemented in the FB p3 program are mainly based upon the Breakthrough method, developed by the Institute of Healthcare Improvement (IHI) (Kilo, 1998; IHI, 2003). This method stands for a very structured way of establishing changes in a short period of time. The goal is to improve health care quality significantly by using methods that have been proven successfully: creating a breakthrough. Within that philosophy, teams set local goals and report on indicators that are of local relevance. Collecting data on a team level targeted at local priorities is inherently a part of the breakthrough method. Therefore the idea of a database with data on centrally defined indicators was thought to fit badly with the 'Breakthrough philosophy' adapted for the majority of the projects. Using standardized key-indicators was felt to corrupt the idea that all quality improvements must be fit towards local circumstances and needs [12,21]. This clash of philosophies was especially apparent within the consortium itself, leading to contradicting communications on the importance of data management.

### Action

In most breakthrough programs the participating teams develop their own data management system and spreadsheet. This is seen as an element of the breakthrough method. However, to insure that all teams would adopt the same standardized indicators, the FB p3 data management team provided all teams in the hospitals with prefab spreadsheets. The teams used the spreadsheets and the central data management indicators proved to be applicable to each team. Although a few teams deviated from the central indicators because of local factors, in general standardization within the breakthrough method worked well.

Also, the importance of the data management project was discussed at length with project leaders and hospital advisors in monthly meetings. Whereas some traces of the clash of the philosophies remained, most of this withered away during the first year of the program.

### Time path

The start of the FB p3 program was forwarded in time. Therefore the database was built simultaneously with the startup of all the other projects. As a result, there was not enough time to test all indicators in a real life situation. In a few cases this resulted in problems with gathering data for the database later on.

### Actions

Imperfections from the first series were learned from and used as lessons in later series. In the first series in the first year the spreadsheets were introduced and handed out to the teams before the QI projects started. This meant that a lot of teams were already collecting data before they actually were told for what purpose they were filling out the spreadsheets. In the second year, teams could be better instructed. Also, for the second and third round of hospitals a special meeting was organized three months before the actual start of the program to inform program coordinators of all the things they should have in place before the start. Data management was an explicit part of these meetings, and hospitals were advised to creating a supporting structure for data collection, both concerning possible IT solutions and having assistants for teams to help in data collection.

### Program communication

In line with the progressive startup of the FB p3 program the communication within the program was at times incomplete. As a result, many people were at first not aware of the function of the database. It was considered a foreign body and not an integral part of the FB p3 program. Many teams were not informed on the existence of the database at all. Surprisingly when all was settled it appeared that the teams had been working without any significant complaints with the standardized data forms designed for data management purposes. The biggest bottleneck then occurred at retrieving data from the teams in the hospitals. The initial lack of communication resulted in stagnation of data traffic from the teams to the database. Teams sometimes didn't know where to send their data. There were cases in which secretaries gathered data that never reached the database. There was more data stuck in the twilight zone, than there was in the database during the first year.

### Actions

The best way to communicate that there were gaps in the database appeared to be periodic feedback reports to the teams. The teams were presented with the available data in the feedback reports and were asked whether this presented a truthful image of their results for the stakeholders in the quarterly progress reports. They would get another two weeks to complete the data when necessary. At first, feedback reports were sent to the hospitals directly. As this created much negative feedback, in further rounds it was decided to send the feedback reports to project leaders of the Breakthrough projects first. This allowed them to complement the database with data they or the secretaries got from the projects. Only after this, the reports were sent to the hospitals. The feedback reports made the teams feel the urgency to complete the data and inspired the teams to be more accurate in delivering their data to the database. The confrontation with empty cells where results were gained made clear that the database could also be a positive way to communicate progress, or at least the effort they put into it to the stakeholders, if only data were send in. Secretaries were also instructed to send incoming data to the database.

In the second and third cohort the hospitals were better prepared. New hospitals to the FB p3 program were advised to install a data management desk. This desk would have a central role in data traffic from the teams to the database.

### Final discussion

A number of qualitative and quantitative goals were set at the start of the FB p3 program. The quantitative goals for the FB p3 program were operationalized as key-indicators provided by standardized data. Using standardized data seemed to be in conflict with the Nolan cycle underlying the FB p3 program and the Breakthrough methodology. Other barriers for central data management were the sensitivity of the data and the political climate at the start of the FB p3 program causing severe mistrust in the participating hospitals. The jumpstart made by the FB p3 program, leaving too little time for piloting the indicators and introducing the database made it even harder to retrieve data from the teams working at the FB p3 program. Admitting to these problems, a lot of effort was put into communication. The database was systematically brought into every meeting to be discussed at each level of the program. Results from the teams were reported back in periodical progress reports to make the teams aware that the data in the database would be used as an indicator for the progress of the FB p3 program as a whole. Even though not all teams were aware of it, data has been collected from the onset of the program due to the spreadsheets that were handed out at the kick-of of the projects. The biggest challenge in the first year was retrieving all data to the central database.

An important methodological factor complicating both gathering and analyzing the data was lack of control. The FB p3 program, like other QICs, was not set up as a research project. The purpose of working in an experimental design is that all confounding factors would be controlled for and data would be standardized. Instead the FB p3 program is a quality improvement program. This means that it is not only impossible but also undesirable to control for al processes and factors. Within a controlled setting, processes do not deviate from plan and if they do all factors influencing the process are accounted for as much as possible. Within the FB p3 program processes are altered all the time as a part of the quality improvement process [22]. The basic thought underlying the FB p3 program was the Nolan Cycle [23]. This cycle is based upon the principle of plan-do-check-act, implying that improvement continues and starts all over again and again improving upon earlier results. This is fundamentally different from a controlled research design in which it is undesirable to improve upon the design during the experiment. Moreover, the Nolan cycle, as well as the Breakthrough method, work from the assumption that project goals are set reflecting local ambitions and circumstances, meaning that goals can (or even should) differ between project teams. Setting fixed quality indicators for aggregated evaluation and benchmarking purposes works against this philosophy. Within the FB p3 program it was decided to gather both qualitative and quantitative data to monitor progress. Eventually gathering somewhat standardized key indicators in a central database is a pragmatic solution to generate quantitative results. Introducing a database within the FB p3 program in this sense can be seen an intervention in itself as it forces project teams and hospitals more generally to work on a set of common goals, making performance measurable and visible. In Table 3 the most essential recommendations for QICs during the design phase are given based on the experienced described in this paper.

**Table 3:** Summary of recommendations for designing QIT's

Communication	Communicate on all levels, both management as care workers in the teams. Create a transparent design in which each person understands his purpose. Make sure that the QIT design is presented well before onset.
Data traffic	When there is data traffic make clear that it must be secured. Medical data must always be encrypted when it leaves the hospital even when it is anonymous.
Security	Data must be stored in a secure environment, preferably with a third party organization specialized in storing medical data. Ownership of the data must be subjected in a data management protocol.
Spreadsheet	Working with standardized spreadsheets leads to standardized data. Working with spreadsheets is not in line with the philosophy of the breakthrough method. In large QITs it is however inevitable to have at least part of the data standardized. By providing spreadsheets that are easy to extend with other variables it can even help promote additional data gathering.
Feedback	Central data management sometimes only seems to create demands for the teams working in QITs. It's therefore essential for data management to provide the teams with valuable feedback on different levels, both on their own teams, as their own hospital as the project they are involved in. Providing useful feedback encourages the teams to deliver their data to the central database.
Confidence	Quality improvement is about people and their positions. Their must be absolute confidence on how the data is trafficked, stored and on how the results are used. People will not cooperate in a process of which they think it might harm their position or institutions. A data management protocol with rules and regulations on handling the data and ownership of the data can be helpful in creating confidence.
Data management desk	Only a few of the hospitals involved in the FB p3 program had a tradition on standardized and large-scale data management. In the first cohort unfamiliarity with data management was an obstacle. The hospitals in the second cohort were advised to install a data management desk that could assist all participating teams within the hospital with their data traffic.

One obstacle that has not been met in the Faster Better collaborative, and is questionable to be met in any QIC, is that project teams themselves acquire data. This leaves all evaluation based on self report. It is questionable whether this strategy will lead to valid results [3]. Only by being aware of the processes described in this paper the results in the database of the FB p3 program can be used for monitoring and evaluation purposes. When used for external purposes it remains necessary to use both qualitative and quantitative results. Only in that way will it be possible to describe the results of the program in the right context. Evaluating the extremely complex projects that QICs are, thus calls for evaluation methods that do justice to this complexity. Gathering quantitative data however will be part of that endeavor and the experiences with data management in a QIC as expressed in this communication will help in creating better data.

## Conclusion

In this paper we have reported on data management within the Faster Better improvement collaborative in the Netherlands, in which 24 hospitals with 515 teams have participated in improving patient safety and logistics, leadership and patient centeredness. A number of issues have been central to data management within this QIC: overcoming resistance to the sharing and publication of data, enabling registration on not normally registered performance, and the tensions between improvement and evaluation and research. Communicating the role of data at all levels of the program, securing data, providing teams with standardized data sheets and sending regular updates on data collection have increased response to comparatively high levels.

### Recommendations

The recommendations coming from this study are:

▪ From our experience it is clear that quality improvement programs deviate from experimental research in many ways. It is not only impossible, but also undesirable to control processes and standardize data streams. QIC's need to be clear of data protocols that do not allow for change. It is therefore minimally important that when quantitative results are gathered, these results are accompanied by qualitative results that can be used to correctly interpret them.

▪ Monitoring and data acquisition interfere with routine. This makes a database collecting data in a QIC an intervention in itself. It is very important to be aware of this in reporting the results. Using existing databases when possible can overcome some of these problems but is often not possible given the change objective of QICs.

▪ Introducing a standardized spreadsheet to the teams is a very practical and helpful tool in collecting standardized data within a QIC. It is vital that the spreadsheets are handed out before baseline measurements start.

## Competing interests

Mireille van den Berg coordinated data management within the FB p3 program during the period this paper refers to. Rianne Frenken was data manager and Roland Bal was a member of the board of the Better Faster QIC.

## Authors' contributions

MB and RB made a first set-up of the article; MB made a first draft; all authors contributed in further refinements.

## Appendix I

### Quantitative goals of the FB p3 program

Patient logistics goals:

- Admission times for the policlinics is reduced to less than one week

- Passage time reduction by 40-90%

- Increase of productivity on the OR by 30%

- Reduction of length of stay by 30%

Patient safety goals:

- Introduction of the blame free reporting system

- Reduction of the number of medication errors by 50%

- Reduction of pressure ulcers to a level under 5%

- Reduction of postoperative wound infections by 50%

- Reduction of postoperative pain to a mean of < 4 on the VAS scale

## Pre-publication history

The pre-publication history for this paper can be accessed here:


